# Optical coherence tomography angiography findings of the fellow eye of patients with unilateral neovascular age-related macular degeneration OCT-A Evaluation of Fellow Eyes of CNV

**Published:** 2019

**Authors:** Sinan Emre, Mahmut Oğuz Ulusoy

**Affiliations:** * Department of Ophthalmology, Başkent University Zübeyde Hanım Research Hospital, İzmir, Turkey; ** Department of Ophthalmology, Başkent University School of Medicine, Konya Research Hospital, Konya, Turkey

**Keywords:** choroidal neovascularization, drusen, optical coherence tomography angiography, pigment epithelial detachment

## Abstract

**Purpose.** Age related macular degeneration (ARMD) remains a serious cause of vision loss in elderly people worldwide. The purpose of the study is to investigate the fellow eye of the patients with exudative ARMD using optical coherence tomography angiography (OCTA).

**Methods.** We conducted a retrospective study from the data of patients with exudative ARMD. Patients undergoing intravitreal anti-VEGF treatment for one eye were selected. OCTA images of both eyes with choroidal neovascularization (CNV) and the fellow eyes were evaluated retrospectively. Midchoroid, choriocapillaris (CC), retina pigment epithelium (RPE), and outer retina levels were evaluated using RTVue XR AVANTI OCTA.

**Results.** There were 5 male and 5 female patients included in this study. We detected drusen and pigment epithelial detachments (PED) in fellow eyes. Six fellow eyes had vascularized PED. Four of them were acute flat irregular type; two of them were chronic dome shaped type. Four patients had soft drusen that had shown signal loss on choriocapillaris level.

**Conclusion.** The follow-up of both eyes of the patients with ARMD is very important. OCTA seems a promising non-invasive method in order to detect subclinical early stage CNV and distinguish among types of PEDs.

## Introduction

Age related macular degeneration (ARMD) is one of the significant reason of irreversible vision loss in adults over the age of 50 years in developed countries [**1**,**2**]. The ARMD has been divided into two subtypes as neovascular or non-neovascular, according to the presence of choroidal neovascularization (CNV) [**3**]. Three types of CNV have been identified. Type 1 is typically placed under retinal pigment epithelium (RPE) and related with a pigment epithelial detachment (PED). Type 2 is present above the RPE in the subretinal layer. Initial location of type 3 is intraretinal [**4**]. Type 1 and 3 constitute the majority of neovascular ARMD cases [**5**]. In a recent study, the authors reported that most fellow eyes had signs of early ARMD, scarring was started with 34.6% of fellow eyes. [**6**]. Therefore, the situation and the follow-up of these eyes became very serious.

Indocyanine green angiography (ICGA) and fluorescein angiography (FA) are effective methods for detecting CNV, however, these techniques require using intravenous contrast agent and side effects including anaphylaxis can be seen with these agents [**7**,**8**]. Although, CNV associated subretinal, intraretinal fluid and/ or PED are effectively seen with optical coherence tomography (OCT); these devices do not allow a direct visualization of neovascular network [**9**]. Optical coherence tomography angiography (OCTA) is a novel method to evaluate the retinal and choroidal vascular layers without the need for contrast agent [**10**,**11**]. 

The efficacy of the OCTA on diagnosis of CNV was shown in previous studies [**5**,**9**,**12**]. The early detection of the CNV on fellow eyes may become more important. In this study, we aimed to evaluate the fellow eyes of patients with CNV using OCTA.

## Methods

This retrospective study was performed on fellow eyes of patients with CNV who were under intravitreal anti-VEGF treatment were evaluated using OCTA, retrospectively. This study adhered to the tenets of the Declaration of Helsinki. Informed consent was obtained from each patients. Due to the retrospective nature, the IRB/ Ethics Committee ruled that an approval was not required for the study.

Patients, whose have exudative neovascular ARMD in one eye and non-exudative ARMD in the fellow eye documented by drusen and/ or RPE changes. All patients underwent total ophthalmic examination including: best corrected visual acuity assessment, anterior segment evaluation with slit lamp, intraocular pressure measurement by goldmann applanation tonometry, fundoscopy with dilatation, FA, OCT, and OCTA.

RTVue XR AVANTI; Optovue Inc, Fremont, CA were used to obtain images. The AngioVue OCTA system was operated at 70,000 A scans per second. The system uses a light source centralized on 840 nm and a bandwidth of 50 nm. The OCTA volumes included 304 · 304 Ascans with 2 consecutive Bscans. Split-spectrum amplitude-decorrelation angiography (SSADA) was used to obtain the OCTA data [**13**]. Every OCTA volume was achieved over 3 seconds, and 2 orthogonal OCTA volumes were achieved to make motion correction to decrease the motion artifacts. [**14**]. The modifications in reflectivity are directly related to blood flow.

OCTA was also capable of visualizing PED through semi-automated segmentation of the outer retina and subretinal or sub-RPE space by using a volumetric SD-OCT data set. OCTA software was used to automatically split the retinal layers and was able to remove retinal vessel shadowing by subtracting vessels seen above the outer plexiform layer from the outer retina OCTA image. Additionally, OCTA images were manually segmented into four layers as superficial capillary plexus, deep capillary plexus, outer retina (to analyze CNV), and choriocapillaris. The outer border of each segment was then individually setted to align with Bruch membrane. This time-intensive method of segmentation ensured accurate determination of the vessel depth within the OCTA scan, thus allowing a more precise differentiation of CNV from choroidal vasculature and a more detailed analysis of CNV characteristics. Qualitative analysis of the OCTA images were conducted by two masked clinicians (M.O. Ulusoy and S. Emre), at different times. 

## Results

**Fig. 1:**
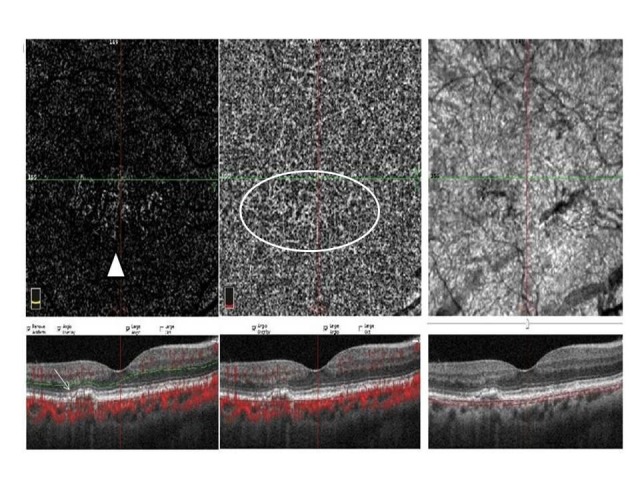
This image shows outer retina level and choriocapillaris level of OCTA (Optical coherence tomography angiography) scan and an OCT (Optical coherence tomography) scan. Acute flat irregular vascularized PED (pigment epithelial detachment) is seen on OCT image (white arrow) and neovascular network is seen at outer retina (white arrowhead) and choriocapillaris level (white circle) on OCTA image

**Fig. 2:**
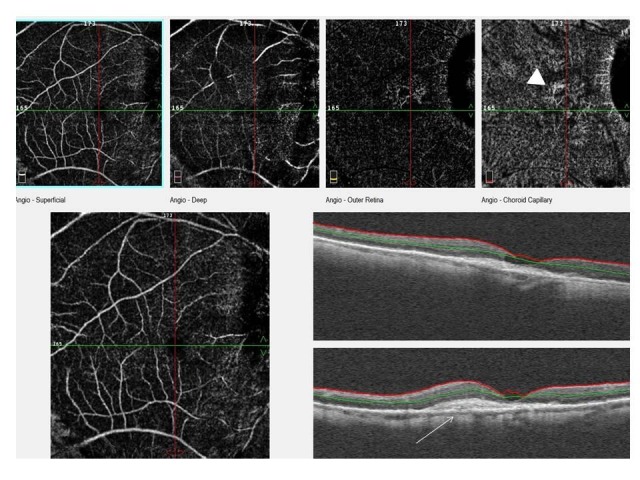
This image shows the superficial and deep capillary level, outer retina level and choriocapillaris level of OCTA scan and an OCT scan. Chronic dome shaped vascularized PED is seen on OCT image (white arrow) and neovascularization is seen at choriocapillaris level on OCTA image (white arrowhead)

**Fig. 3:**
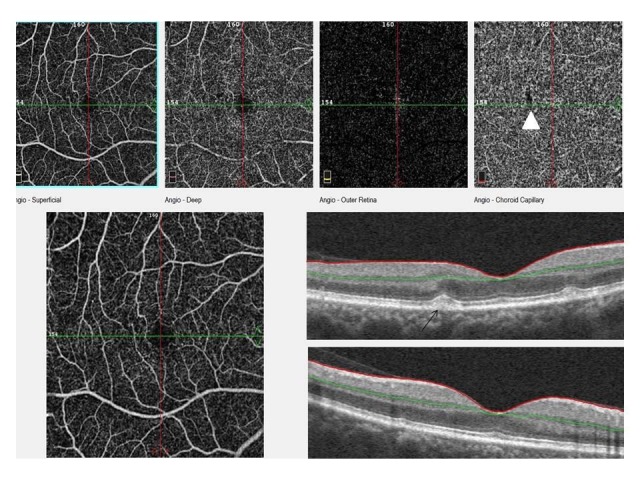
This image shows the superficial and deep capillary level, outer retina level and choriocapillaris level of OCTA scan and an OCT scan. Drusenoid PED is seen on OCT image (black arrow) and signal loss of drusen area is seen at choriocapillaris level on OCTA (white arrowhead)

**Fig. 4:**
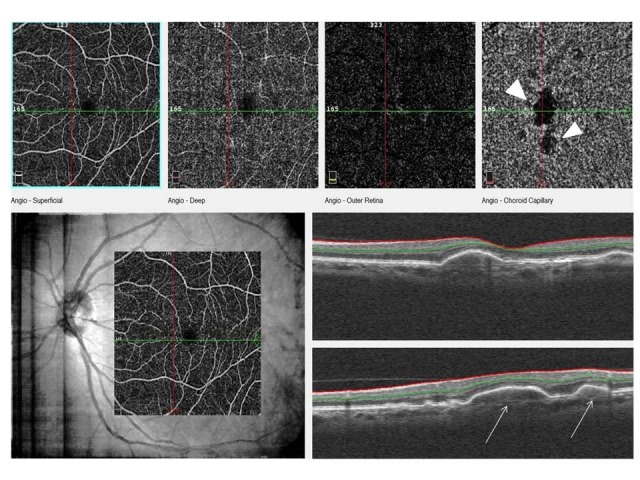
This image shows the superficial and deep capillary level, outer retina level and choriocapillaris level of OCTA scan and an OCT scan. Serous PED on OCT image (white arrows) and large signal loss area is seen at choriocapillaris level on OCTA image (white arrowheads)

Twenty eyes of 10 patients were evaluated (mean age was 72±9.33 years; 5 males and 5 females) in this study. Each patient’s eye had CNV shown with FA and the fellow eye did not. Seven of the CNV were type 1 and 3 of them were type 2. There were 4 sea fan type CNV, 2 medusa type and 1 indistinct type in eyes with CNV (**Table 1**). In two fellow eyes, more than one type pigment epithelial detachment (PED) was found. We detected vascularized PED in 6 fellow eyes. Four of them were acute flat irregular type (**Fig. 1**), two of them were chronic dome shaped type (**Fig. 2**). Four of the eyes had drusenoid PED (**Fig. 3**) and 2 of them had serous PED (**Fig. 4**). In addition, 4 patients had soft drusen that had shown signal loss on choriocapillaris level. 

**Table 1:** OCTA characteristics of CNV and fellow eyes of the patients

Patients	Gender	Age	CNV Eye	CNV type	Fellow Eye
1	M	67	Sea fan	1	Drusenoid PED
2	M	67	Sea fan	1	Drusenoid PED
3	F	72	Sea fan	1	Acute Flat Irregular Vascularized PED
4	F	77	Medusa	1	Serous PED
5	M	52	Indistinct	1	Chronic Dome Shaped Vascularized PED, Drusenoid PED, Serous PED
6	F	69		2	Acute Flat Irregular Vascularized PED
7	F	79		2	Serous PED
8	F	73	Sea fan	1	Acute Flat Irregular Vascularized PED
9	M	87	Medusa	1	Acute Flat Irregular Vascularized PED
10	M	77		2	Chronic Dome Shaped Vascularized PED
OCTA = Optical coherence tomography angiography, CNV = choroidal neovascularization, PED = pigment epithelial detachment,					

## Discussion

Early diagnosis and treatment of CNV is crucial to ensure superior visual outcomes. Therefore, it is noteworthy to evaluate and differentiate early changes of ARMD. Drusen and PED are often seen in early and intermediate ARMD [**15**,**16**]. However, FA and OCT are effective methods for evaluating drusen and PED, having some limitations [**17**]. OCTA is a noninvasive technique for rapidly acquiring images of the vascular layers of the retina and choroid without any contrast agent injection [**18**]. 

A previous study revealed that type 1 CNV is characterized with a minor demarcation from choroidal vessels and visible on the slab mid-choroid and choriocapillaris (CC); however, type 2 has good demarcation from choroidal vessels and visible on the slab outer retina [**18**]. According to these classifications, 7 of the CNV were type 1 and 3 of them were type 2 in our study. Kuehlewein et al. reported two different type 1 membrane morphologies as medusa and sea fan [**4**]. In our study, 4 of the type 1 CNV were sea fan type and 3 of them were medusa type.

Extracellular deposits which accumulate between the RPE and the inner collagenous layer of Bruch’s membrane are named drusen[**19**]. The drusen and pigmentary changes on the retina are characteristic findings of early and intermediate ARMD [**20**]. The increase of drusen size is as a sign for progression toward advanced stages of ARMD [**21**]. In previous studies, it has been suggested that the site of drusen formation is not random and they are presumably to form at sites of deficiant choroidal perfusion [**16**,**22**]. Therefore, visualization of CC under drusen is noteworthy for probable CNV or geographic atrophy development. In OCTA, false signal loss can be seen on CC slab under drusen. Lane et al. reported that swept-source OCTA is less prone to producing areas of false-positive flow impairment under drusen than in spectral domain OCTA [**17**]. In our study, drusen were seen in 4 of the patients’ fundus examinations. These patients’ fellow eyes needed careful evaluation in regular examinations with OCTA to avoid false positive signal loss.

PED is a pathological process in which the RPE separates from the underlying Bruch’s membrane [**23**]. In ARMD, PED can be classified into drusenoid, serous, vascularized, or mixed types. Drusenoid and serous PEDs are characteristics of nonneovascular ARMD. In contrast, vascularized PEDs are associated with Type 1 CNV [**24**]. 

It is noteworthy to differentiate the vascularized PED from other types, using non-invasive methods especially. In a previous study, authors reported that OCTA could differentiate the PEDs from each other [**24**]. Vascularized PEDs were classified according to OCT images as acute flat irregular and chronic dome shaped. Acute flat irregular vascularized PEDs were associated with monolayered CNV on OCTA, however chronic dome-shaped vascularized PEDs were shown as multilayered CNV on OCTA. In a previous study, it was noted that CNV of chronic vascularized PED appeared multilayered in OCT [**25**]. In our study, 6 of the fellow eyes had vascularized PED. 4 of them were acute flat irregular and 2 of them were chronic dome shaped PED. These vascularized PEDs did not show any sign of CNV with FA that was applied on the same day. In a recent case series, the authors reported subclinical CNV in three asymptomatic fellow eyes of patients [**26**]. A previous similar study reported that they have identified a 6.25% rate of CNV with no clinical signs of exudative ARMD using OCTA [**27**]. In a recent study, the authors detected subclinical CNV 14.4% (23/ 160) of asymptomatic patients with SS-OCTA and 13 of them turned to exudative ARMD [**28**]. Also in another study that evaluated the vessel densities, choroidal thickness and vascular tortuosity of the CNV eye, asymptomatic fellow eye and controls, it was reported that vessel densities of CNV and fellow eyes were significantly different from controls; however, they did not differ among themselves [**29**]. In addition, they did not evaluate subclinical CNV in the fellow eyes. 

In conclusion, in this study we evaluated fellow eyes of patients with CNV using with OCTA. OCTA can differentiate vascularized PEDs from serous and drusenoid PEDs. The most important point is that non-exudative CNVs can be revealed with OCTA. We suggested that the follow-up of the fellow eye of patients with ARMD should be done carefully and in short intervals using OCTA, which is a novel non-invasive method. 

**Competing/ conﬂicts of interest**

No conﬂict of interest stated.

**Funding sources**

No funding sources stated.
